# Supplementary material for: The rare sugar d-tagatose protects plants from downy mildews and is a safe fungicidal agrochemical
Source: Commun Biol. 2020 Aug 5;3:423. doi: 10.1038/s42003-020-01133-7 (PMC7406649; doi:10.1038/s42003-020-01133-7)
Supplement: Supplementary file 1 — Supplementary Information [file 42003_2020_1133_MOESM1_ESM.pdf]

Supplementary Information

Supplementary Figure 1

Supplementary Figure 2

Supplementary Figure 3

Supplementary Figure 4

Supplementary Figure 5

Supplementary Figure 6

Supplementary Table 1

Supplementary Table 2

Supplementary Table 3

Supplementary Table 4

Supplementary Methods

Supplementary Figures

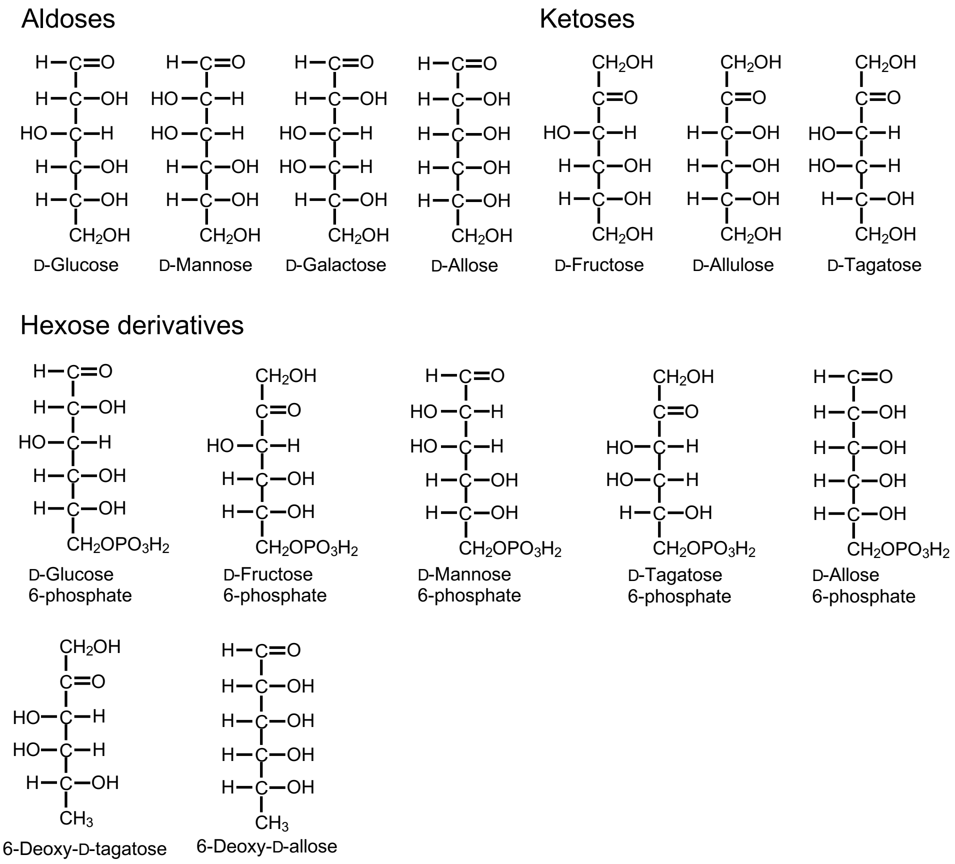

Supplementary Fig. 1 Fisher projections of various hexoses and their derivative structures described in this study

Four common sugars (D-glucose, D-fructose, D-mannose, D-galactose), three rare sugars (D-allulose, D-allose, D-tagatose), and the sugar derivatives (D-glucose 6-phosphate, D-fructose 6-phosphate, D-mannose 6-phosphate, D-tagatose 6-phosphate, D-allose 6-phosphate, 6-deoxy-D-tagatose, 6-deoxy-D-allose) that are described in this work are shown.

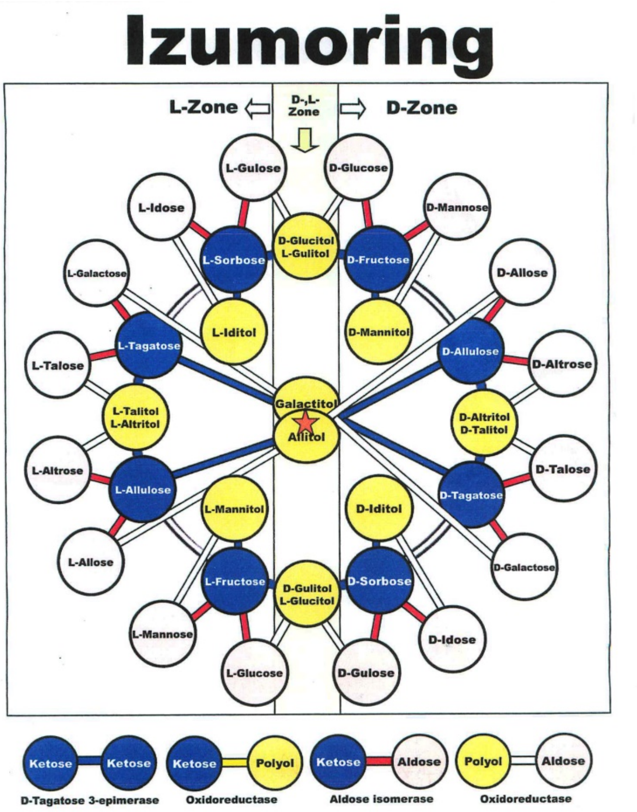

Supplementary Fig. 2 Blueprint to enzymatically synthesize all hexoses including 30 rare-sugar hexoses based on the Izumoring concept from 2002[2, 3]

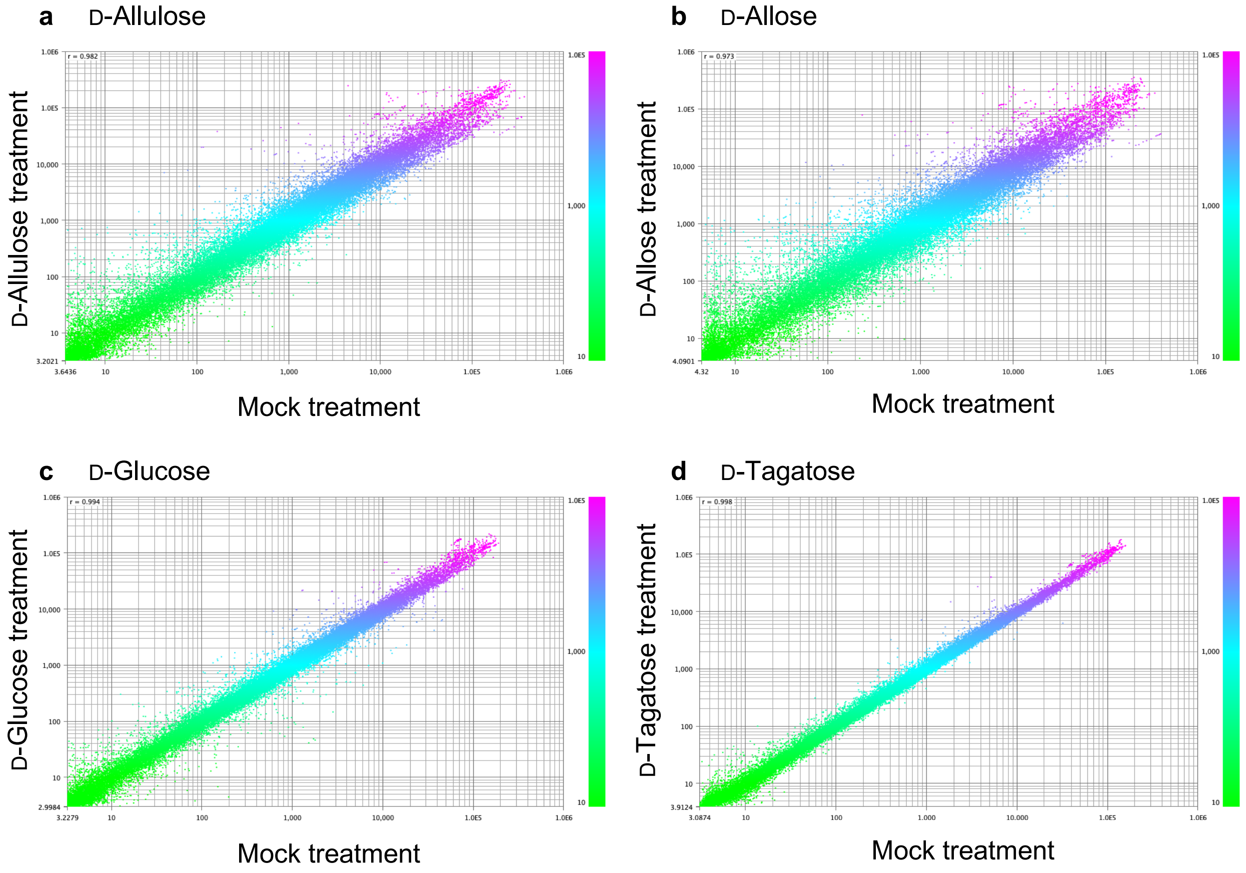

Supplementary Fig. 3 Scatter plots of measurements for microarray analyses of various sugar-treated plants of *Oryza sativa*

O. sativa seedlings were cultured in Kimura B broth containing 0.5 mM of D-allulose[14] (**a**), D-allose[16, 17] (**b**), D-glucose[14, 16, 17] (**c**), D-tagatose (**d**) or no sugar for 2 days. Total RNA was labeled with Cy3 or Cy5 following hybridization and washing according to the manufacturer's instructions (Agilent Technologies, Redwood City, CA, USA), and hybridized microarrays were then scanned with an Agilent Microarray Scanner as described previously[14, 16, 17]. The microarray analyses were done three times. Scatter plots were analyzed using the Subio platform 1.22.5473 (Subio, Amami, Japan). All data used for these scatter plots were deposited as data files in the Gene Expression Omnibus (GEO) Database with accession of GSE19595 for D-allulose[14], GSE15479 for D-allose[16, 17] and D-glucose[14, 16, 17], and GSE136313 for D-tagatose for the rice.

a

LOCUS ID	GENE NAME	log2 fold_change	*q*-value
**JA/ET pathway**			
AT3G12500	CHITINASE (CHIB), PATHOGENESIS-RELATED 3 (PR3)	0.256011	0.999751
AT3G04720	PATHOGENESIS-RELATED 4 (PR4)	0.758725	0.809764
AT5G44420	PDF1.2a / LCR77	-0.943227	0.945559
AT2G26020	PDF1.2b	0.880354	0.990807
AT3G45140	LIPOXYGENASE 2 (LOX2)	-0.087581	0.999751
AT5G24770	VEGETATIVE STORAGE PROTEIN 2 (VSP2)	0.551700	0.999751
AT1G19180	JAZ1	-1.083140	0.792862
AT1G17380	JAZ5	-1.321010	0.796513
AT5G13220	JAZ10	0.315980	0.999751
AT1G61120	GERANYLLINALOOL SYNTHASE (GES / TPS4)	-1.167350	0.861099
AT1G19670	CORONATINE-INDUCED PROTEIN 1 (ATCLH1),	0.861557	0.839742
AT3G23240	ETH RESPONSE FACTOR 1 (ERF1)	1.266010	0.824115
AT1G06160	OCTADECANOID-RESPONSIVE ARABIDOPSIS AP2 / ERF 59 (ORA59 / ERF59)	-0.216375	0.999751
AT5G03280	ETHYLENE INSENSITIVE 2 (EIN2)	0.151679	0.999751
AT3G20770	ETHYLENE INSENSITIVE 3 (EIN3)	-0.312011	0.999751
**SA pathway**			
AT2G14610	PATHOGENESIS-RELATED 1 (PR1)	0.001389	0.999751
AT3G57260	PATHOGENESIS-RELATED 2 (PR2)	0.611036	0.940003
AT1G75040	PATHOGENESIS-RELATED 5 (PR5)	0.226335	0.999751
AT3G48090	EDS1	0.145031	0.999751
AT1G74710	EDS16 / ISOCHORISMATE SYNTHASE 1 (ICS1)	0.929566	0.792862
AT5G06950	TGA2	0.306771	0.999751
AT5G06960	TGA5 / OBF5	-0.309324	0.999751
AT3G12250	TGA6	0.176094	0.999751
**Cell wall modifications**			
AT4G18780	CELLULOSE SYNTHASE A (IRX1 / CESA8 / LEW2)	0.562238	0.999751
AT5G17420	CELLULOSE SYNTHASE A (IRX3 / CESA7)	0.581395	0.999751
AT5G44030	CELLULOSE SYNTHASE A (IRX5 / CESA4)	0.842028	0.971514
AT5G06860	POLYGALACTURONASE INHIBITOR PROTEIN (PGIP1)	-0.395782	0.999751
AT5G06870	POLYGALACTURONASE INHIBITOR PROTEIN (PGIP2)	-0.137190	0.999751
AT1G11580	PECTIN METHYLESTERASE PCR A (ATPMEPCRA)	0.384011	0.999751
**MAMPs response**			
AT3G21630	Chitin Elicitor Receptor Kinase 1 (CERK1)	-0.164757	0.999751
AT2G17120	Chitin oligosaccharides binding protein (AtCEBiP / LYM2)	-0.017813	0.999751
AT1G21880	Peptide glycan binding protein (LYM1)	0.905485	0.938717
AT1G77630	Peptide glycan binding protein (LYM3)	0.775013	0.970538
AT5G46330	FLAGELLIN-SENSITIVE 2 (FLS2)	0.013788	0.999751
AT4G32430	BRI1-ASSOCIATED RECEPTOR KINASE (BAK1)	0.175969	0.999751

b

Group 1: Upregulated Arabidopsis genes in D-tagatose-treated Arabidopsis innoculated with Hpa (≥10 times)

ORF	DESCRIPTION	Gene Symbol	Gene group	Fold change	q-value
AT2G14247	unknown expressed protein.			56.72	0.0021
AT2G27550	TERMINAL FLOWER LIKE 1 (TFL1) like protein.	ATC	regulation	30.04	0.7929
AT2G41240	basic helix-loop-helix protein 100.	BHLH100	regulation	26.99	0.5937
AT2G14245	copia-like retrotransposon family			26.63	0.7929
AT3G18590	EARLY NODULIN-like protein 5.	AtENODL5		21.15	0.7929
AT3G56980	basic helix-loop-helix protein 39, OBP3-responsive protein 3 (ORG3).	BHLH039	growth regulation	18.68	0.7929
AT5G45830	DELAY OF GERMINATION 1 (DOG1).	DOG1	sugar sensing	18.66	0.7929
AT5G40900	Nucleotide-diphospho-sugar transferase family protein.			17.07	0.7929
AT2G14230	CACTA-like transposase family (Ptta/En/Spm).			15.94	0.7929
AT3G56970	basic helix-loop-helix protein 38, OBP3-responsive protein 3 (ORG3).	BHLH038	growth regulation	15.25	0.7929
AT2G33100	cellulose synthase-like protein.	ATCSLD1	glucosyltransferase	14.57	0.7929
AT2G02430	Arginyl-tRNA synthetase.			14.34	0.7929
AT1G15840	hypothetical protein.			14.34	0.7929
AT1G48660	Auxin-responsive GH3 family protein.		plant hormone-related	13.66	0.7929
AT1G13609	defensin-like (DEFL) family protein.			12.82	0.7929
AT3G59250	F-box/RNI-like superfamily protein.			12.63	0.7929
AT5G50800	SWEET sucrose efflux transporter family proteins.	SWEET13	sugar sensing	12.63	0.7929
AT5G04150	basic helix-loop-helix protein 101.	BHLH101		12.55	0.7929
AT2G30900	TRICHOME BIREFRINGENCE LIKE 43 (TBL43) protein.	TBL43		12.43	0.9998
AT1G69140	hypothetical protein.			11.76	0.7929
AT5G45820	CBL-INTERACTING PROTEIN KINASE 20 (CIPK20).	CIPK20	stress response	11.29	0.7929
AT1G24580	RING/U-box superfamily protein.			11.26	0.7929
AT3G43715	hypothetical protein.			10.92	0.7929
AT5G13655	hypothetical protein.			10.24	0.7929

Group 2: Downregulated Arabidopsis genes in D-tagatose-treated Arabidopsis innoculated with Hpa (≤1/10 times)

ORF	DESCRIPTION	Gene Symbol	Gene group	Fold change	q-value
AT2G15555	unknown other RNA			0.034	0.7929
AT3G45120	transposable_element.			0.040	0.7929
AT2G41850	Arabidopsis dehiscence zone polygalacturonase 2.	ADPG2	polygalacturonase	0.050	0.7929
AT2G20800	NAD(P)H dehydrogenase B4, NDB4	NDB4	oxidative stress response	0.051	0.7929
AT5G55135	hypothetical protein			0.068	0.7929
AT2G01422	unknown other RNA			0.085	0.7929
AT5G43570	PR(pathogenesis-related)-6 proteinase inhibitor family.		pathogenesis-related	0.088	0.7929
AT1G03670	Ankyrin repeat containing protein.			0.096	0.7929
AT1G61475	ATP binding / protein kinase.			0.097	0.7929
AT3G09220	putative laccase	LAC7	phenoloxidase	0.098	0.7929

Group 3: Upregulated Hpa genes in D-tagatose-treated Arabidopsis innoculated with Hpa (≥10 times)

ORF	DESCRIPTION	Gene Symbol	Gene group	Fold change	q-value
HpaG810197	hypothetical protein			9155.50	0.9998
HpaG802161	hypothetical protein			336.79	0.9998
HpaG814340	hypothetical protein			13.12	0.9998

Group 4: Downregulated Hpa genes in D-tagatose-treated Arabidopsis innoculated with Hpa (≤1/10 times)

ORF	DESCRIPTION	Gene Symbol	Gene group	Fold change	q-value
HpaG801065	Family 30 glycoside hydrolase		β-glucosylceramidase	0.00002	0.9998
HpaG804978	hypothetical protein			0.0003	0.9998
HpaG803006	hypothetical protein			0.037	0.7929
HpaG813039	hypothetical protein			0.049	0.7929
HpaG801662	SET and MYND domain-containing protein		histone modification	0.050	0.9998
HpaG809727	hypothetical protein			0.055	0.9998
HpaG811686	Tetratricopeptide TPR1			0.057	0.7929
HpaG807708	Nep1-like protein			0.061	0.7929
HpaG800089	RxLR effector candidate protein		effector	0.061	0.7929
HpaG804565	hypothetical protein			0.062	0.7929
HpaG810405	hypothetical protein			0.062	0.7929
HpaG803132	hypothetical protein			0.067	0.9998
HpaG805184	reverse transcriptase			0.068	0.7929
HpaG814630	hypothetical protein			0.068	0.7929
HpaG803976	hypothetical protein			0.070	0.8680
HpaG805561	hypothetical protein			0.071	0.7929
HpaG802119	C2 domain-containing protein 3			0.074	0.9998
HpaG811639	hypothetical protein			0.075	0.7929
HpaG804913	hypothetical protein			0.075	0.7929
HpaG804576	hypothetical protein			0.075	0.7929
HpaG804454	hypothetical protein			0.076	0.7929
HpaG802958	hypothetical protein			0.078	0.9998
HpaG805908	hypothetical protein			0.080	0.7929
HpaG813824	hypothetical protein			0.083	0.9998
HpaG807989	hypothetical protein			0.084	0.7929
HpaG803884	Ankyrin repeat containing protein			0.085	0.7929
HpaG803599	RxLR effector candidate protein		effector	0.085	0.7929
HpaG807922	hypothetical protein			0.091	0.7929
HpaG814386	hypothetical protein			0.092	0.7929
HpaG810932	hypothetical protein			0.096	0.7929
HpaG809220	hypothetical protein			0.097	0.7929
HpaG814787	hypothetical protein			0.097	0.7929
HpaG808139	hypothetical protein			0.097	0.7929
HpaG805821	Leucine rich repeat containing protein			0.097	0.7929
HpaG810684	hypothetical protein			0.097	0.7929

Supplementary Fig. 4 RNA-seq for *A. thaliana* inoculated with *Hyaloperonospora arabidopsidis* isolate Noco2 after D-tagatose treatment

Total RNA from *A. thaliana* inoculated with isolate Noco2 with D-tagatose was compared with those without D-tagatose treatment by RNA-seq analyses. The log2 fold changes of the respective data in Fig. 3c were shown with *q*-values (**a**). All genes up- or downregulated more than 10 times are listed above by classifying as respective groups; Groups 1 and 2 for genes from *A. thaliana*, and Groups 3 and 4 for genes from *H. arabidopsidis* isolate Noco2, and the fold changes of the normalized expression were shown with *q*-value (**b**). Statistical significance (*q*-value) of the comparison was calculated using Cuffdiff (v2.0.2) program[54] as described in Methods.

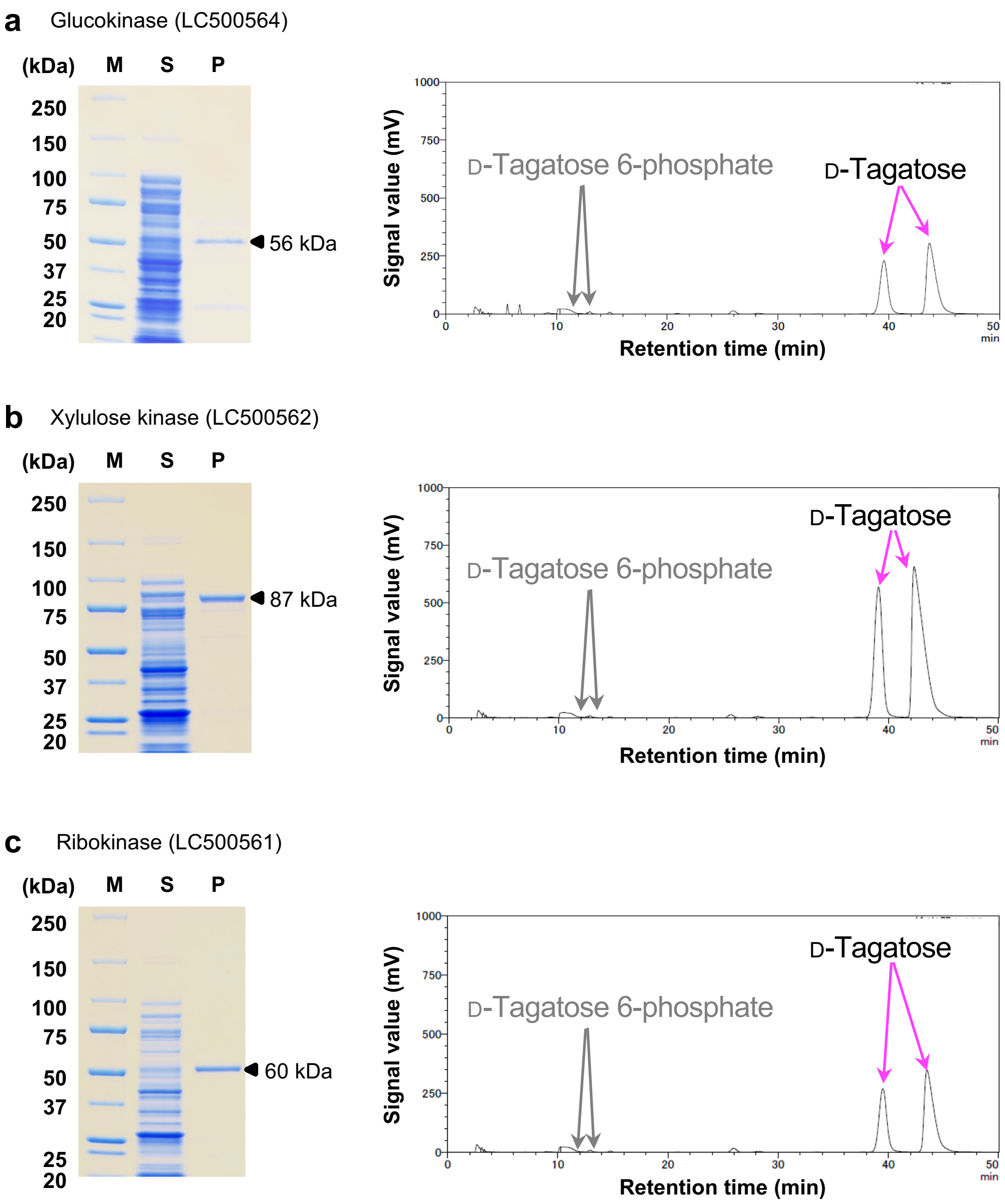

Supplementary Fig. 5 Heterologous expression products and their respective ability to phosphorylate D-tagatose at the C-6 position for four putative genes from *Hyaloperonospora arabidopsidis* isolate Noco2 that were annotated as encoding enzymes with possible function for sugar phosphorylation

The proteins encoded by four putative genes selected from the genomic database were expressed in *E. coli* to test their enzymatic activity and included glucokinase (LC500564 equivalent to Hpa800730 of *H. arabidopsidis* strain Emoy2) (**a**), xylulose kinase (LC500562 equivalent to Hpa801075) (**b**), ribokinase (LC500561 equivalent to Hpa805763) (**c**), and galactokinase (equivalent to Hpa809752), respectively. The gene encoding galactokinase (Hpa809752) was not expressed well in the *E. coli* system (data not shown).

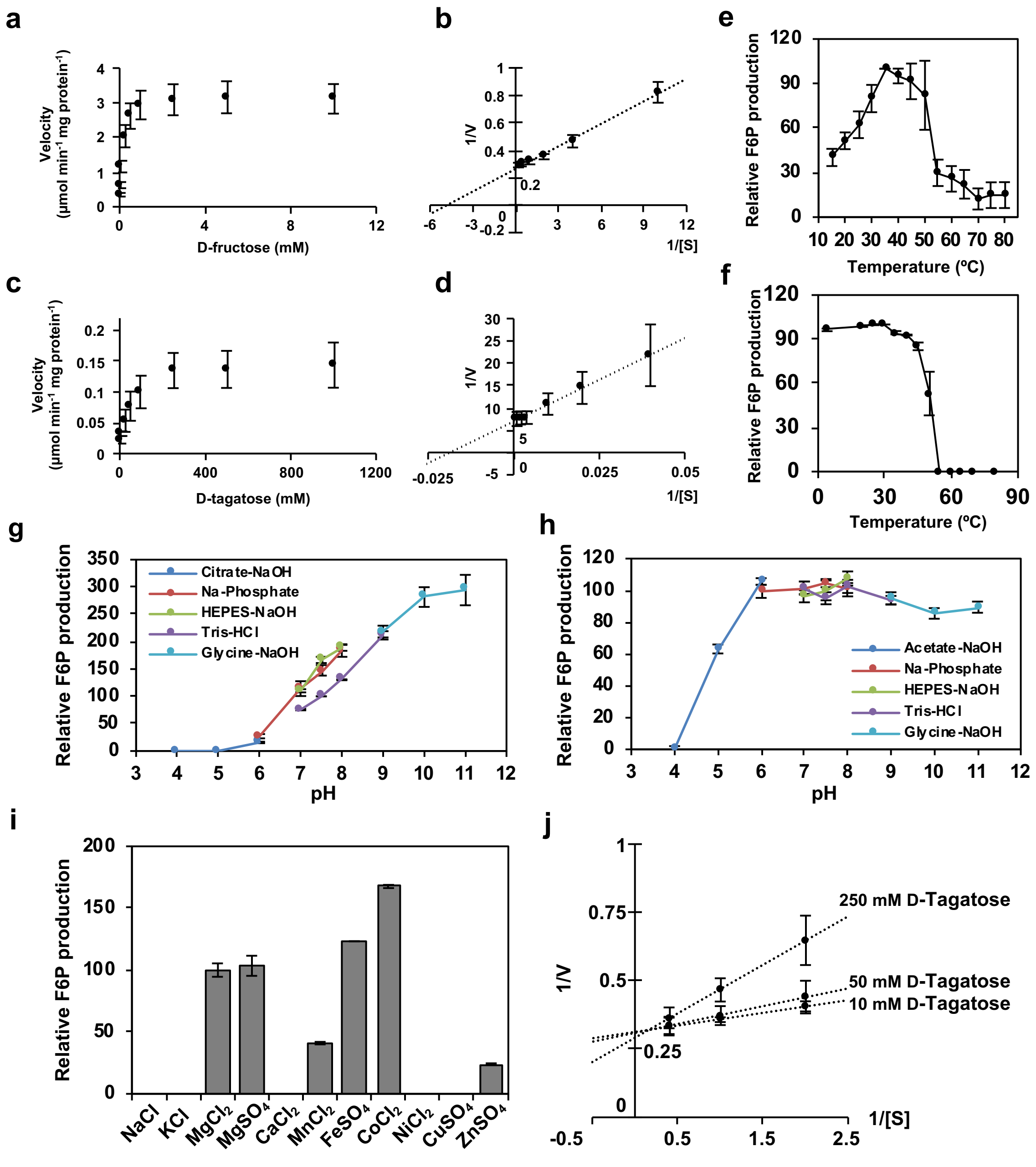

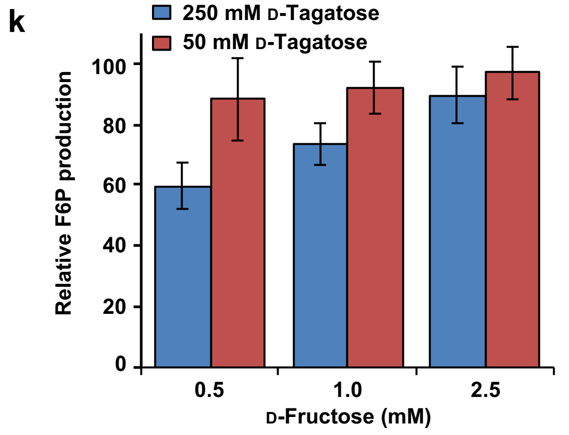

Supplementary Fig. 6 Characterizations of enzymatic properties for fructokinase (LC500344) from *Hyaloperonospora arabidopsidis* isolate Noco2

Michaelis-Menten (**a**) and Lineweaver-Burk (**b**) plots for D-fructose; Michaelis-Menten (**c**) and Lineweaver-Burk (**d**) plots for D-tagatose; optimal temperature (**e**); thermal stability (**f**); optimal pH (**g**); pH stability (**h**); optimal cofactor (metal ion; 2 mM each) (**i**) for D-fructose; Lineweaver-Burk plot of D-fructose with D-tagatose as a competitor (**j**), and calculated inhibitory effect of D-tagatose (50 or 250 mM) on the fructokinase reaction for D-fructose 6-phosphate (F6P) production using 0.5 to 2.5 mM D-fructose based on the data from Fig. 4d (**k**), respectively. Error bars are SD.

Supplementary Tables

Supplementary Table 1 Experimental conditions to test D-tagatose effects against various diseases on different hosts in pot trials

Class	Disease	Pathogen	Host	Experimental design	Stage treated	Concentration (inoculum)	Spray volume	Time of inoculation	Incubation: temperature, duration	Assessment (days after inoculation)	Severity on leaves [a]
Oomycetes	Downy mildew	*Plasmopara viticola*	Grapevine (NeoMuscat)	1 pot, 2 replications	3 to 4 leaves	1 x 10^4 (sporangia)	5 ml pot^{-1} (400 l per 10 a)	1 day after D-tagatose	20°C, 24 h	7 days, 20 to 22°C	0 to 3 rating
		Pseudoperonospora cubensis	Cucumber (Sagami Hanshiro)	1 pot, 2 replications	1 true leaf	8 x 10^3 to 1 x 10^4(sporangia)	5 ml pot^{-1} (400 l per 10 a)	1 day after D-tagatose	20°C, 24 h	7 to 10 days, 20 to 22°C	0 to 3 rating
		Hyaloperonospora parasitica	Cabbage (Shikidori)	1 pot, 2 replications	3 leaves	8 x 10^3 to 1 x 10^4(conidia)	5 ml pot^{-1} (400 l per 10 a)	1 day after D-tagatose	20°C, 24 h	7 to 10 days, 20 to 22°C	0 to 3 rating
	Damping off	*Pythium aphanidermatum*	Cucumber (Sagami Hanshiro)	5 seeds pot-1, 2 replications	seed	100 g l^{-1} soil (mycelia)	5 ml pot^{-1} (3 l m^{-2})	-	-	14 days, 20 to 22°C	Number of diseased seedllings
	Seedling blight	*Pythium graminicola*	Rice (Koshihikari)	25 m2 2 replications	seed	100 g l^{-1} soil (mycelia)	10 ml pot^{-1} (500 ml box^{-1})	-	-	14 days, 20 to 22°C	Percentage of diseased area in pot
	Late blight	*Phytophthora infestans*	Tomato (Ogata Fukujyu)	1 pot, 2 replications	4 to 5 leaves	5 x 10^3 to 1 x 10^4 (sporangia)	5 ml pot^{-1} (400 l per 10 a)	1 day after D-tagatose	20°C, 24 h	5 to 7 days, 20 to 22°C	0 to 3 rating
Ascomycetes	Powdery mildew	*Erysiphe necator*	Grapevine (NeoMuscat)	1 pot, 2 replications	3 leaves	1 x 10^5 (conidia)	5 ml pot^{-1} (400 l per 10 a)	1 day after D-tagatose	-	7 to 10 days, 22 to 23°C	0 to 3 rating
		Podosphaera xanthii	Cucumber (Sagami Hanshiro)	1 pot, 2 replications	1 true leaf	1 x 10^5 (conidia)	5 ml pot^{-1} (400 l per 10 a)	1 day after D-tagatose	-	7 to 10 days, 22 to 23°C	0 to 3 rating
		Podosphaera leucotricha	Apple (Ohrin)	1 pot, 2 replications	3 leaves	1 x 10^5 (conidia)	5 ml pot^{-1} (400 l per 10 a)	1 day after D-tagatose	-	10 days, 22 to 23°C	0 to 3 rating
		Sphaerotheca fuliginea	Eggplant (Mizunasu)	1 pot, 2 replications	3 to 4 leaves	1 x 10^5 (conidia)	5 ml pot^{-1} (400 l per 10 a)	1 day after D-tagatose	-	7 to 10 days, 22 to 23°C	0 to 3 rating
		Oidium violae	Tomato (Ogata Fukujyu)	1 pot, 2 replications	4 to 5 leaves	1 x 10^5 (conidia)	5 ml pot^{-1} (400 l per 10 a)	1 day after D-tagatose	-	10 to 14 days, 22 to 23°C	0 to 3 rating
		Oidiopsis sicula	Pepper (Kyo Midori)	1 pot, 2 replications	4 to 5 leaves	1 x 10^5 (conidia)	5 ml pot^{-1} (400 l per 10 a)	1 day after D-tagatose	-	20 days, 22 to 23°C	0 to 3 rating
		Blumeria graminis f.sp. *hordei*	Barley (Sekishinriki)	1 pot, 2 replications	2 leaves	dusting (conidia)	5 ml pot^{-1} (400 l per 10 a)	1 day after D-tagatose	-	7 days, 22 to 23°C	0 to 3 rating
	Gray mold	*Botrytis cinerea*	Tomato (Ogata Fukujyu)	1 pot, 2 replications	4 to 5 leaves	1 x 10^6 (conidia)	5 ml pot^{-1} (400 l per 10 a)	1 day after D-tagatose	23°C, 48 to 72 h	2 to 3 days, 23°C	0 to 3 rating
	Altenaria	*Alternaria brassicicola*	Cabbage (Shikidori)	1 pot, 2 replications	cotyledon	1 x 10^6 (conidia)	5 ml pot^{-1} (400 l per 10 a)	1 day after D-tagatose	23°C, 48 to 72 h	2 to 3 day, 23°C	0 to 3 rating
	Brown spot	*Cochliobolus miyabeanus*	Rice (Sachikaze)	1 pot, 2 replications	2.5 leaves	1 x 10^4 (conidia)	5 ml pot^{-1} (400 l per 10 a)	1 day after D-tagatose	28°C, 24 h	7 days, 22 to 23°C	0 to 3 rating
	Anthracnose	*Colletotrichum orbiculare*	Cucumber (Sagami Hanshiro)	1 pot, 2 replications	1 true leaf	1 x $10^{6 \text{ to } 7}$ (conidia)	5 ml pot^{-1} (400 l per 10 a)	1 day after D-tagatose	23°C, 24 h	7 to 10 day, 22 to 23°C	0 to 3 rating
	Blast	*Pyricularia oryzae*	Rice (Sachikaze)	1 pot, 2 replications	2.5 leaves	1 x 10^6 (conidia)	5 ml pot^{-1} (400 l per 10 a)	1 day after D-tagatose	23°C, 24 h	10 days, 20 to 22°C	0 to 3 rating
Basidiomycetes	Brown rust	*Puccinia recondita*	Wheat (Norin 61)	1 pot, 2 replications	2 leaves	1 x 10^5 (urediospores)	5 ml pot^{-1} (400 l per 10 a)	1 day after D-tagatose	23°C, 24 h	7 to 10 days, 20 to 22°C	0 to 3 rating
	Sheath blight	*Rhizoctonia solani* AG-1 IA	Rice (Sachikaze)	1 pot, 2 replications	2.5 leaves	8 oat grains pot^{-1} (mycelia)	5 ml pot^{-1} (400 l per 10 a)	1 day after D-tagatose	28°C, 24 h	7 to 10 days, 23 to 25°C	0 to 3 rating

[a] Rating of 0 to 3: 0, no symptoms; 0.1, diseased area 3%; 0.3, 10% diseased area; 0.8, 25% diseased area; 1.5, 50% diseased area; 2, 70% diseased area; 3, 95% or more diseased area

Supplementary Table 2 Experimental conditions to test D-tagatose effects on diseases of different hosts in field trials

Class	Disease	Pathogen	Host Plant	Site (Pref., Japan)	Experimental Design	Stage (BBCH)[52]	Application Date	Spray Volume	Inoculation (Concentration)	Assessment Date	Disease Rating	Disease Severity
Oomycete	Downy mildew	*Plasmopara viticola*	Grapevine (Kyohou)	Shiga	1/2 tree, 3 replications	19 to 55	2009/6/9, 16, 24	300 l per 10 a	2009/6/11 (sporangia $1x10^{3 \text{ to } 4}$)	2009/7/1	0, no symptoms; 1, diseased area <10%; 2, diseased area 11 to 30%; 3, diseased area 31 to 50%; 4, diseased area ≥51%	$100 \times \{(1n_1+2n_2+3n_3+4n_4)/4N\}$; N = 50, n = number of leaves each disease rating
		Pseudoperonospora cubensis	Cucumber (Ancor 8)	Shiga	8 plants, 3 replications	19 to 62	2009/6/19, 26, 7/3	300 l per 10 a	2009/6/22 (sporangia $1x10^{3 \text{ to } 4}$)	2009/7/10	0, no symptoms; 1,diseased area <5%; 2, diseased area 5 to <25%; 3, diseased area 25 to <50% 4, diseased area ≥50%	$100 \times \{(1n_1+2n_2+3n_3+4n_4)/4N\}$; N = 50, n = number of leaves each disease rating
		Hyaloperonospora parasitica (syn. *H. brassicae*)	Chinese cabbage (Kigokoro 75)	Miyagi	20 plants, 3 replications (8 leaves/plant)	45 to 49	2010/10/11, 18, 24	250 to 300 l per 10 a	Natural infection	2010/10/31	0, no symptoms; 1, Nubmer of symptom 1 to 10; 2, Nubmer of symptom 11 to 20; 3, Nubmer of symptom ≥21	$100 \times \{(1n_1+2n_2+3n_3)/3N\}$; N = 160, n = number of leaves each disease rating
		Peronospora destructor	Onion (Gifuki)	Gifu	50 plants, 3 replications	41 to 48	2010/4/14, 19, 29	200 l per 10 a	Natural infection	2010/5/8	0, no symptoms; 1,diseased area <5%; 2, diseased area 5 to <25%; 3, diseased area 25 to <50%; 4, diseased area ≥50%	$100 \times \{(1n_1+2n_2+3n_3+4n_4)/4N\}$; N = 50, n = number of leaves each disease rating
		Peronospora farinosa f.sp. *spinaciae*	Spinach (Jiromaru)	Gifu	3.6 m^2, 20 plants, 3 replications (7 to 8 leaves)	15 to 19	2010/3/25, 4/1	230 l per 10 a	2010/2/13 (infested soil)	2010/4/8	0, no symptoms; 1, diseased area <1/3; 2, diseased area 1/3 to <2/3; 3, diseased area ≥2/3; 4, diseased area 100 death	$100 \times \{(1n_1+2n_2+3n_3+4n_4)/4N\}$; N = 140 to 163, n = number of leaves each disease rating
	Seedling blight	*Pythium graminicola*	Rice (Koshihikari)	Shiga	nursery box, 3 replications	7 to 12	2008/3/25	500 l per box	100 g l^{-1} oil (mycelium)	2008/4/4	diseased area in nursery box	–
	Late blight	*Phytophthora infestans*	Potato (Nishiyutaka)	Nagasaki	3.25 m^2, 20 plants, 3 replications	102 to 115	2010/4/8, 15, 23, 30, 5/7	150 to 250 l per 10 a	2010/4/8, 15 (diseased leaf)	2010/5/7, 13	0, no symptoms; 1, diseased area 1/4; 2, diseased area 1/2; 3, diseased area almost diseased; 4, diseased area death	$100 \times \{(1n_1+2n_2+3n_3+4n_4)/4N\}$; N = 20, n = number of leaves each disease rating
Ascomycete	Powdery mildew	*Podosphaera xanthii*	Cucumber (Ancor 8)	Shiga	5 plants, 2 replications	19 to 62	2009/6/19, 26, 7/3	300 l per 10 a	Natural infection	2009/7/10	0, no symptoms; 1,diseased area <5%; 2, diseased area 5 to <25%; 3, diseased area 25 to <50%; 4, diseased area ≥50%	$100 \times \{(1n_1+2n_2+3n_3+4n_4)/4N\}$; N = 50, n = number of leaves each disease rating
		Podosphaera aphanis	Strawberry (Sagahonoka)	Saga	10 plants, 3 replications	55 to 92	2010/6/16, 23, 30	300 l per 10 a	Natural infection	2010/7/14, 7/21	0, no symptoms; 1,diseased area <5%; 2, diseased area 5 to <25%; 3, diseased area 25 to <50%; 4, diseased area ≥50%	$100 \times \{(1n_1+2n_2+3n_3+4n_4)/4N\}$; N = 30, n = number of leaves each disease rating
		Sphaerotheca fuliginea	Eggplant (Mizunasu)	Shiga	6 plants, 2 replications	61 to 71	2011/4/18, 28, 5/19	300 l per 10 a	Natural infection	2011/5/26	0, no symptoms; 1,diseased area <5%; 2, diseased area 5 to <25%; 3, diseased area 25 to <50%; 4, diseased area ≥50%	$100 \times \{(1n_1+2n_2+3n_3+4n_4)/4N\}$; N = 50, n = number of leaves each disease rating
		Oidiopsis sicula	Pepper (Kyo Midori)	Shiga	6 plants, 3 replications	61 to 71	2010/6/7, 15, 25, 7/5	300 l per 10 a	Natural infection	2010/7/16	0, no symptoms; 1,diseased area <5%; 2, diseased area 5 to <25%; 3, diseased area 25 to <50%; 4, diseased area ≥50%	$100 \times \{(1n_1+2n_2+3n_3+4n_4)/4N\}$; N = 50, n = number of leaves each disease rating
Basidiomycete	Brown rust	*Puccinia recondita*	Wheat (Norin 61)	Shiga	5 m^2, 3 replications	47 to 65	2009/5/19, 27	150 l per 10 a	Natural infection	2009/6/12	0, no symptoms; 1, Number of uredinia 1 to 5; 2, 6 to 12; 3, 13 to 25; 4, 26 to 50; 5, 51 to 100; 6, ≥100	$100 \times \{(1n_1+2n_2+3n_3+4n_4+5n_5+6n_6)/6N\}$; N = 90, n = number of leaves each disease rating

N = number of leaves in one replication

Supplementary Table 3 Summary of cDNA clones identified from subtraction library of mock- (no sugar) and D-tagatose-treated cucumber leaves

Clone ID	Homologous Gene	Length (bp)	Score	Expected Value
cuc32412-1A04	ABK55715.1 purple acid phosphatase-like protein [*Cucumis sativus*]	164	276	1.00E-72
cuc32412-1A07	CAO23933.1 unnamed protein product [*Vitis vinifera*]	476	250	7.00E-65
cuc32412-1A08	ACF80095.1 unknown [*Zea mays*]	228	278	2.00E-73
cuc32412-1A09	CAO42829.1 unnamed protein product [*Vitis vinifera*]	1008	197	4.00E-49
cuc32412-1A10	NP_564916.2 CCoAMT (caffeoyl-CoA 3-*O*-methyltransferase) [*Arabidopsis thaliana*]	232	111	5.00E-23
cuc32412-1A12	BAC23052.1 putative lipid transfer protein [*Solanum tuberosum*]	93	50.1	1.00E-04
cuc32412-1B04	AAZ81836.1 isoamylase isoform 2 [*Pisum sativum*]	857	107	1.00E-21
cuc32412-1B06	AAA79186.1 lipoxygenase [*Cucumis sativus*]	877	180	9.00E-44
cuc32412-1B08	ACF79064.1 unknown [*Zea mays*]	377	172	1.00E-41
cuc32412-1B10	CAN61244.1 hypothetical protein [*Vitis vinifera*]	430	101	5.00E-20
cuc32412-1B11	CAO24159.1 unnamed protein product [*Vitis vinifera*]	433	77.4	8.00E-13
cuc32412-1B12	NP_564352.1 AGL79 (AGAMOUS-LIKE 79) [*Arabidopsis thaliana*]	97	59.7	2.00E-07
cuc32412-1C01	ABD96966.1 hypothetical protein [*Cleome spinosa*]	218	138	3.00E-31
cuc32412-1C02	CAO40399.1 unnamed protein product [*Vitis vinifera*]	1193	39.7	0.19
cuc32412-1C03	CAO24546.1 unnamed protein product [*Vitis vinifera*]	363	149	1.00E-34
cuc32412-1C04	ABS72194.1 RSH2 [*Corchorus olitorius*]	239	340	4.00E-92
cuc32412-1C06	AAX35343.1 glutamine synthetase [*Cucumis melo*]	432	237	6.00E-61
cuc32412-1C10	AAO84915.1 galactinol synthase [*Cucumis sativus*]	331	428	1.00E-118
cuc32412-1C12	AAW48295.1 pore-forming toxin-like protein Hfr-2 [*Triticum aestivum*]	492	60.5	1.00E-07
cuc32412-1D01	CAO48676.1 unnamed protein product [*Vitis vinifera*]	183	131	4.00E-29
cuc32412-1D03	NP_174692.1 unknown protein [*Arabidopsis thaliana*]	657	202	1.00E-50
cuc32412-1D04	AAW48295.1 pore-forming toxin-like protein Hfr-2 [*Triticum aestivum*]	492	75.5	3.00E-12
cuc32412-1D06	CAO15188.1 unnamed protein product [*Vitis vinifera*]	531	70.1	1.00E-10
cuc32412-1D08	AAA33128.1 peroxidase [*Cucumis sativus*]	294	207	4.00E-52
cuc32412-1D09	AAX18166.2 CNGC2 [*Gossypium hirsutum*]	715	140	8.00E-32
cuc32412-1D10	XP_001988168.1 GH10714 [*Drosophila grimshawi*]	1920	36.2	2.1
cuc32412-1D11	NP_177257.3 phosphatidylinositol-4-phosphate 5-kinase family protein [*Arabidopsis thaliana*]	1648	261	2.00E-68
cuc32412-1D12	ABX09988.1 cullin 4 [*Solanum lycopersicum*]	785	235	2.00E-60
cuc32412-1E02	CAN75238.1 hypothetical protein [*Vitis vinifera*]	657	147	5.00E-34
cuc32412-1E04	CAF18247.1 SEU1 protein [*Antirrhinum majus*]	841	61.2	6.00E-08
cuc32412-1E05	ABK94768.1 unknown [*Populus trichocarpa*]	319	137	7.00E-31
cuc32412-1E06	NP_173562.1 2-oxoisovalerate dehydrogenase. putative / 3-methyl-2-oxobutanoate dehydrogenase. putative / branched-chain alpha-keto acid dehydrogenase E1 alpha subunit. putative [*Arabidopsis thaliana*]	472	63.2	6.00E-09
cuc32412-1E07	CAO63491.1 unnamed protein product [*Vitis vinifera*]	444	92.4	2.00E-17
cuc32412-1E08	BAD46002.1 unknown protein [*Oryza sativa* japonica group]	695	47.4	0.001
cuc32412-1E09	CAO38724.1 unnamed protein product [*Vitis vinifera*]	180	134	6.00E-30
cuc32412-1E10	CAN80536.1 hypothetical protein [*Vitis vinifera*]	471	191	4.00E-47
cuc32412-1E11	YP_001971992.1 putative amino-acid transporter transmembrane protein [*Stenotrophomonas maltophilia* K279a]	491	35	3.4
cuc32412-1F01	CAN67215.1 hypothetical protein [*Vitis vinifera*]	523	76.6	1.00E-12
cuc32412-1F02	CAO24546.1 unnamed protein product [*Vitis vinifera*]	363	155	3.00E-36
cuc32412-1F04	EAY89742.1 hypothetical protein OsI_010975 [*Oryza sativa* indica group]	411	46.2	0.001
cuc32412-1F05	ABB89042.1 glutathione reductase [*Vigna unguiculata*]	499	207	5.00E-52
cuc32412-1F07	CAN70560.1 4-Coumarate-CoA ligase (4CL) [*Vitis vinifera*]	663	198	2.00E-49
cuc32412-1F08	CAO15405.1 unnamed protein product [*Vitis vinifera*]	815	88.2	5.00E-16
cuc32412-1F09	CAO68040.1 unnamed protein product [*Vitis vinifera*]	467	78.2	5.00E-13
cuc32412-1F10	CAQ39629.1 transporter. putative [*Plasmodium knowlesi* strain H]	2518	35.4	3.6
cuc32412-1F11	AAX26850.2 SJCHGC01954 protein [*Schistosoma japonicum*]	128	35.4	3.5
cuc32412-1G03	CAO60961.1 unnamed protein product [*Vitis vinifera*]	539	166	1.00E-39
cuc32412-1G04	ABK93931.1 unknown [*Populus trichocarpa*]	224	217	7.00E-55
cuc32412-1G05	AAX35343.1 glutamine synthetase [*Cucumis melo*]	432	237	6.00E-61
cuc32412-1G07	CAO62858.1 unnamed protein product [*Vitis vinifera*]	877	43.1	0.017
cuc32412-1G08	CAO67982.1 unnamed protein product [*Vitis vinifera*]	440	155	3.00E-36
cuc32412-1G10	CAO39905.1 unnamed protein product [*Vitis vinifera*]	466	112	2.00E-23
cuc32412-1H01	NP_193816.1 FAD-binding domain-containing protein [*Arabidopsis thaliana*]	539	168	3.00E-40
cuc32412-1H03	CAN70158.1 hypothetical protein [*Vitis vinifera*]	237	335	1.00E-90
cuc32412-1H04	CAN75238.1 hypothetical protein [*Vitis vinifera*]	657	85.1	5.00E-30
cuc32412-1H07	CAO15188.1 unnamed protein product [*Vitis vinifera*]	531	68.6	4.00E-10
cuc32412-1H10	ABK95396.1 unknown [*Populus trichocarpa*]	358	221	4.00E-56
cuc32412-1H12	CAO60867.1 unnamed protein product [*Vitis vinifera*]	1088	325	1.00E-87
cuc32412-62A01	NP_567466.1 unknown protein [*Arabidopsis thaliana*]	256	80.9	7.00E-14
cuc32412-62A02	YP_002291029.1 hypothetical protein p1056.15 [*Haemophilus influenzae*]	159	61.2	6.00E-08
cuc32412-62A03	CAO15188.1 unnamed protein product [*Vitis vinifera*]	531	70.1	1.00E-10
cuc32412-62A05	CAN60668.1 hypothetical protein [*Vitis vinifera*]	771	44.7	0.006
cuc32412-62A06	CAO61507.1 unnamed protein product [*Vitis vinifera*]	604	169	1.00E-40
cuc32412-62B01	ABK95585.1 unknown [*Populus trichocarpa*]	267	124	7.00E-27
cuc32412-62B02	CAO62123.1 unnamed protein product [*Vitis vinifera*]	343	77.8	6.00E-13
cuc32412-62B03	AF169022_1 seed maturation protein PM37 [*Glycine max*]	417	120	1.00E-25
cuc32412-62B04	AF317062_1 abscisic stress ripening-like protein [*Prunus persica*]	193	41.6	0.049
cuc32412-62B05	CAO22822.1 unnamed protein product [*Vitis vinifera*]	232	34.7	5.8
cuc32412-62B06	ACG36734.1 hydrolase. alpha/beta fold family protein [*Zea mays*]	491	170	9.00E-41
cuc32412-62C01	CAD10148.1 phosphoenolpyruvate carboxylase [*Cucumis sativus*]	198	92	3.00E-17
cuc32412-62C02	EAW57562.1 hCG2004566 [*Homo sapiens*]	107	33.9	9.7
cuc32412-62C03	CAO61740.1 unnamed protein product [*Vitis vinifera*]	450	142	2.00E-32
cuc32412-62C05	CAO45516.1 unnamed protein product [*Vitis vinifera*]	823	57.8	7.00E-07
cuc32412-62D01	XP_001617664.1 hypothetical protein NEMVEDRAFT_v1g69851 [*Nematostella vectensis*]	138	22.3	1.5
cuc32412-62D02	CAN74265.1 hypothetical protein [*Vitis vinifera*]	506	125	2.00E-27
cuc32412-62D03	CAO71073.1 unnamed protein product [*Vitis vinifera*]	241	64.7	5.00E-09
cuc32412-62D04	XP_567747.1 hypothetical protein CNK02440 [*Cryptococcus neoformans* var.*neoformans* JEC21]	136	23.5	0.08
cuc32412-62E01	ACJ86116.1 unknown [*Medicago truncatula*]	130	38.9	0.3
cuc32412-62E02	CAO66198.1 unnamed protein product [*Vitis vinifera*]	850	163	1.00E-38
cuc32412-62E04	CAO62609.1 unnamed protein product [*Vitis vinifera*]	526	42	0.035
cuc32412-62E05	ABK93469.1 unknown [*Populus trichocarpa*]	413	80.9	7.00E-14
cuc32412-62E06	CAO15633.1 unnamed protein product [*Vitis vinifera*]	344	110	1.00E-22
cuc32412-62F01	AAP43511.1 oxygen-evolving enhancer protein 3 precursor [*Pisum sativum*]	234	105	3.00E-21
cuc32412-62F02	CAO66548.1 unnamed protein product [*Vitis vinifera*]	415	129	2.00E-30
cuc32412-62G01	ACJ85858.1 unknown [*Medicago truncatula*]	610	193	7.00E-48
cuc32412-62G02	BAD26571.1 type-2 metallothionein [*Citrullus lanatus*]	77	112	3.00E-23
cuc32412-62G05	CAO21852.1 unnamed protein product [*Vitis vinifera*]	810	137	5.00E-31
cuc32412-62G06	CAO22822.1 unnamed protein product [*Vitis vinifera*]	232	34.7	5.8
cuc32412-62H01	AAM63708.1 submergence induced protein 2A [*Arabidopsis thaliana*]	187	127	6.00E-28
cuc32412-62H03	CAO65492.1 unnamed protein product [*Vitis vinifera*]	323	125	3.00E-27
cuc32412-6A02	ABK93754.1 unknown [*Populus trichocarpa*]	216	211	3.00E-53
cuc32412-6A03	CAN78550.1 hypothetical protein [*Vitis vinifera*]	384	158	3.00E-37
cuc32412-6A05	O98997 ribulose bisphosphate carboxylase/oxygenase activase.chloroplastic [*Vigna radiata* var. *radiata* (Mung bean) (*Phaseolus aureus*)]	439	138	4.00E-31
cuc32412-6A06	CAA98160.1 RAB1C [*Lotus japonicus*]	202	301	2.00E-80
cuc32412-6A07	CAD80088.1 putative major latex protein [*Momordica charantia*]	121	178	3.00E-43
cuc32412-6A08	CAO15188.1 unnamed protein product [*Vitis vinifera*]	531	68.2	5.00E-10
cuc32412-6A11	ACE97168.1 2OG-Fe(II) oxygenase [*Populus tremula*]	117	109	2.00E-22
cuc32412-6A12	ABK93754.1 unknown [*Populus trichocarpa*]	216	231	3.00E-59
cuc32412-6B03	AAK19619.1|AF3362861 GHMYB9 [*Gossypium hirsutum*]	264	45.4	0.003
cuc32412-6B05	EEC84646.1 hypothetical protein OsI_31535 [*Oryza sativa* indica group]	426	34.3	7.9
cuc32412-6B10	ACJ84128.1 unknown [*Medicago truncatula*]	136	106	1.00E-21
cuc32412-6B11	XP_001902255.1 U88 [*Brugia malayi*]	120	26.2	5.5
cuc32412-6B12	Q39677 S-adenosylmethionine decarboxylase proenzyme 2 [*Dianthus caryophyllus*]	377	100	6.00E-43
cuc32412-6C01	CAO23857.1 unnamed protein product [*Vitis vinifera*]	607	216	1.00E-54
cuc32412-6C02	CAO61625.1 unnamed protein product [*Vitis vinifera*]	623	146	1.00E-33
cuc32412-6C05	CAO42829.1 unnamed protein product [*Vitis vinifera*]	1008	196	1.00E-48
cuc32412-6C07	ABD96966.1 hypothetical protein [*Cleome spinosa*]	218	138	3.00E-31
cuc32412-6C08	NP_568107.1 PRR7 (PSEUDO-RESPONSE REGULATOR 7); transcription regulator [*Arabidopsis thaliana*]	727	113	1.00E-23
cuc32412-6C11	ABK93754.1 unknown [*Populus trichocarpa*]	216	80.1	1.00E-13
cuc32412-6D02	CAO49802.1 unnamed protein product [*Vitis vinifera*]	975	124	4.00E-27
cuc32412-6D04	AAL38344.1 unknown protein [*Arabidopsis thaliana*]	793	259	8.00E-68
cuc32412-6D07	O98997 ribulose bisphosphate carboxylase/oxygenase activase.chloroplastic [*Vigna radiata* var. *radiata*]	439	138	3.00E-31
cuc32412-6D09	ABN08705.1 DDT; homeodomain-related [*Medicago truncatula*]	1795	63.2	1.00E-08
cuc32412-6D10	AAA79186.1 lipoxygenase [*Cucumis sativus*]	877	180	8.00E-44
cuc32412-6D11	CAO60961.1 unnamed protein product [*Vitis vinifera*]	539	222	2.00E-56
cuc32412-6E03	CAN63005.1 hypothetical protein [*Vitis vinifera*]	538	181	3.00E-44
cuc32412-6E07	CAO15661.1 unnamed protein product [*Vitis vinifera*]	253	38.5	0.41
cuc32412-6E08	CAN68594.1 hypothetical protein [*Vitis vinifera*]	473	57.8	6.00E-07
cuc32412-6E11	AAM23263.1 DnaJ-like protein [*Glycine max*]	164	122	2.00E-26
cuc32412-6E12	CAO23857.1 unnamed protein product [*Vitis vinifera*]	607	136	8.00E-31
cuc32412-6F02	CAO70007.1 unnamed protein product [*Vitis vinifera*]	503	350	3.00E-95
cuc32412-6F03	CAO42202.1 unnamed protein product [*Vitis vinifera*]	369	68.2	5.00E-10
cuc32412-6F04	ACJ84128.1 unknown [*Medicago truncatula*]	136	107	8.00E-22
cuc32412-6F05	ACF85852.1 unknown [*Zea mays*]	199	37	1
cuc32412-6F06	NP_174692.1 unknown protein [*Arabidopsis thaliana*]	657	202	1.00E-50
cuc32412-6F10	CAO68040.1 unnamed protein product [*Vitis vinifera*]	467	78.2	5.00E-13
cuc32412-6F11	ABK96690.1 unknown [*Populus trichocarpa* x *Populus deltoides*]	148	55.8	2.00E-06
cuc32412-6F12	NP_001052057.1 Os04g0118400 [*Oryza sativa* japonica group)]	843	294	3.00E-78
cuc32412-6G01	CAO41275.1 unnamed protein product [*Vitis vinifera*]	477	58.5	3.00E-07
cuc32412-6G03	AAX35343.1 glutamine synthetase [*Cucumis melo*]	432	213	7.00E-54
cuc32412-6G05	CAO67982.1 unnamed protein product [*Vitis vinifera*]	440	155	3.00E-36
cuc32412-6G06	XP_001621769.1 hypothetical protein NEMVEDRAFT_v1g143700 [*Nematostella vectensis*]	213	24.6	0.12
cuc32412-6G10	ACJ85698.1 unknown [*Medicago truncatula*]	471	96.7	1.00E-18
cuc32412-6H05	CAO65619.1 unnamed protein product [*Vitis vinifera*]	381	281	4.00E-74
cuc32412-6H06	BAD94511.1 hypothetical protein [*Arabidopsis thaliana*]	532	172	2.00E-41
cuc32412-6H07	ABB89042.1 glutathione reductase [*Vigna unguiculata*]	499	202	2.00E-50
cuc32412-6H10	CAN60668.1 hypothetical protein [*Vitis vinifera*]	771	66.2	2.00E-09

Notes: From the sequence analyses of 288 clones, 190 sequences were obtained, and 137 genes and their annotations are given. The other clones either yielded no sequence results or no function could be identified. Colored rows indicate the genes with annotations of putative PR-protein or proteins related to defense used for additional RT-qPCR analyses shown in Fig. 3a.

Supplementary Table 4 Primers used in this study

Primer	Direction	Primer sequence (5' to 3')
RT-qPCR analyses for cucumber gene expression		
cucActin	Forward	gagacattcaatgtgcctgctatg
	Reverse	cacgatatccagtggtacgtcca
cuc32412-1D08 POX	Forward	ccccttggcttccagtta
	Reverse	acgcaaggggctaaaacagt
cuc32412-1B06 LOX	Forward	cggccgaggtacaaag
	Reverse	tcagaagcatgccttgacaat
cuc32412-1C12 Hfr	Forward	aactcggtggggatgaa
	Reverse	tctttcgtttcaccccatgt
cucCU567 PI	Forward	cttgctcctcctgaaa
	Reverse	gacatggccacagttgttctt
cuc32412-1F07 4CL	Forward	ctcttgaatggtgcaacc
	Reverse	tgataaagccggtgaagtcc
cuc32412-1A10 CCoAMT	Forward	ccggataacggcaatt
	Reverse	ttattgagggcaaccaaagc
Construction of *E. coli* expression vectors		
fructokinase (LC500344)	Forward	gaagatcttcatgactcgatacgcagg
	Reverse	ggaattcccttgtgctgctcgagttc
glucokinase (LC500564)	Forward	gaagatcttcatgggatacggtctcctc
	Reverse	ggaattcccttccgcccgagaat
xylulose kinase (LC500562)	Forward	gaagatcttcatgtcaccttcaagtatcgac
	Reverse	ggaattccgctgagtagactcacagcttg
ribokinase (LC500561)	Forward	gaagatcttcatgattcgaagtaggaagcag
	Reverse	ggaattcccaggcctttctctttgag
phosphomannose isomerase (LC500563)	Forward	gagggatccgaattcatgcagaaactccagtgc
	Reverse	gacaagcttgaattccgatgaagataagctttcg

Supplementary Methods

Supplementary Methods

Kinetic parameters (Supplementary Fig. 6a–d)

For calculating K_m and V_{max} values based on the Lineweaver–Burk plots of the Michaelis–Menten equation, activities of fructokinase (LC500344; 10 μg [D-fructose as a substrate] or 50 μg [D-tagatose]) were determined spectrophotometrically with three independent replications at 340 nm at 25°C for 5 min by coupling production of ADP to oxidation of NADH via pyruvate kinase (15 U ml^{-1}) (Oriental Yeast, Tokyo, Japan) and lactate dehydrogenase (10 U ml^{-1}) (Oriental Yeast) reactions as described by Miller and Raines[56] with either 0.025 to 10 mM D-fructose (Supplementary Fig. 6a, b) or 5 to 1000 mM D-tagatose (Supplementary Fig. 6c, d) in 1 ml reaction mixture containing 80 mM Tris-HCl (pH 7.5), NADH (0.3 mM), phosphoenolpyruvate (0.8 mM), ATP (4 mM), and $MgCl_2$ (8 mM). K_m and V_{max} values for each substrate described in the main text were calculated based on the Lineweaver–Burk plots of the Michaeli–Menten equation shown in Supplementary Fig. 6a–d.

Optimal temperature (Supplementary Fig. 6e)

A reaction mixture containing 50 mM glycine-NaOH buffer (pH 10), 10 mM $MgCl_2$, 25 mM ATP, 10 mM D-fructose, 2 μg fructokinase (LC500344) in 200 μl was incubated at different temperatures (15 to 80°C) for 10 min. After the reaction was inactivated by boiling for 5 min, the reaction products were filtered using a 0.22 μm spin column (Millipore, Burlington, MA, USA) and analyzed with an HPLC system (Prominence; Shimadzu, Kyoto, Japan) using a Shodex Asahipak NH2P-50 4E column (4.6 mm ID×250 mm) (Showadenko, Tokyo, Japan) with a 20 min separation at a flow rate of 1.0 ml min^{-1} at 30°C with 100 mM NaH_2PO_4. The peaks were monitored with a refractive index detector (RID-10A, Shimadzu). Activities were calculated from the respective peak area in the HPLC spectrum using a standard curve generated from standards of known concentrations, and relative F6P production at different temperatures was obtained by comparison to the activity at 35°C set as 100. Means ± SD from four independent replications are shown in Supplementary Fig. 6e.

Thermal stability (Supplementary Fig. 6f)

Fructokinase (LC500344) was heat-treated at different temperatures (4 to 80°C) for 10 min with 5 mM Tris-HCl buffer (pH7.5). A reaction mixture containing 50 mM glycine-NaOH buffer (pH 10), 10 mM $MgCl_2$, 25 mM ATP, 2 μg heat-treated fructokinase (LC500344) and 10 mM D-fructose in 200 μl was incubated at 30°C for 10 min. After the reaction was inactivated by boiling for 5 min, the reaction products were filtered and analyzed with the HPLC system described above. Activities were also calculated as described above, and relative F6P production

at different temperatures was obtained by comparison to the activity at 30°C set as 100. Means ± SD from four independent replications are shown in Supplementary Fig. 6f.

Optimal pH (Supplementary Fig. 6g)

A reaction mixture containing 50 mM buffer (trisodium citrate-NaOH at pH 4, 5, or 6; HEPES at pH 7, 7.5, or 8; NaH_2PO_4 at pH 6, 7, 7.5, or 8; Tris-HCl at pH 7, 7.5, 8, or 9; glycine-NaOH at pH 9, 10, or 11), 10 mM $MgCl_2$, 25 mM ATP, 10 mM D-fructose, and 2 μg fructokinase (LC500344) in 200 μl was incubated at 30°C for 10 min. After the reaction was inactivated by boiling for 5 min, the reaction products were filtered, and then analyzed with the HPLC system as described above. The activities were calculated as describe above, and the relative F6P production at each pH was determined by comparison to the activity at pH 7.5 (Tris-HCl) set as 100. Means ± SD from three independent replications are shown in Supplementary Fig. 6g.

pH stability (Supplementary Fig. 6h)

Fructokinase (LC500344; 10 μg) was pretreated with 100 mM buffer (acetate-NaOH at pH 4, 5, or 6; HEPES at pH 7, 7.5, or 8; NaH_2PO_4 at pH 6, 7, 7.5, or 8; Tris-HCl at pH 7, 7.5, 8, or 9; glycine-NaOH at pH 9, 10, or 11) in 10 μl at 30°C for 16 h. Monosaccharide kinase activity of the pretreated fructokinase was determined spectrophotometrically at 340 nm at 25°C for 5 min by coupling production of ADP to oxidation of NADH via pyruvate kinase (15 U ml^{-1}) (Oriental Yeast, Tokyo, Japan) and lactate dehydrogenase (10 U ml^{-1}) (Oriental Yeast) reactions as described by Miller and Raines[53] with 10 mM of D-fructose in 1 ml reaction mixture containing 80 mM Tris-HCl buffer (pH 7.5), NADH (0.3 mM), phosphoenolpyruvate (0.8 mM), ATP (4 mM) and $MgCl_2$ (8 mM), with addition of the pretreated sample (10 μg fructokinase/10 μl). Activities were calculated as relative F6P production at the different ranges of pH in comparison to activity at pH 7.5 (Tris-HCl) set as 100. Means ± SD from four independent replications are shown in Supplementary Fig. 6h.

Optimal cofactor (metal ion) (Supplementary Fig. 6i)

Fructokinase (LC500344; 10 μg) was pretreated with 5 mM ethylenediaminetetraacetic acid (EDTA) in 5 μl (pH8) at 30°C for 3 h. Monosaccharide kinase activity of the chelated fructokinase was determined spectrophotometrically at 340 nm at 25°C for 5 min by coupling production of ADP to oxidation of NADH via pyruvate kinase (15 U ml^{-1}) (Oriental Yeast) and lactate dehydrogenase (10 U ml^{-1}) (Oriental Yeast) reactions as described by Miller and Raines[56] with 10 mM of D-fructose in 1 ml reaction mixture containing 80 mM Tris-HCl (pH 7.5), NADH (0.3 mM), phosphoenolpyruvate (0.8 mM), ATP (4 mM) with 2 mM each of different

metal ions (Mg^{2+} [$MgCl_2$, $MgSO_4$], Mn^{2+} [$MnCl_2$], Ca^{2+} [$CaCl_2$], Fe^{2+} [$FeSO_4$], Co^{2+} [$CoCl_2$], Ni^{2+} [$NiCl_2$], Cu^{2+} [$CuSO_4$], Zn^{2+} [$ZnSO_4$], Na^+ [$NaCl_2$; as a control], and K^+ [KCl; as a control]), with addition of the EDTA-pretreated sample (10 μg fructokinase/5 μl). Relative F6P production with the different metal ions was calculated in comparison to activity with $MgCl_2$ set as 100. Means ± SD from two independent replications are shown in Supplementary Fig. 6i.
