# Supplementary material for: The rare sugar d-tagatose protects plants from downy mildews and is a safe fungicidal agrochemical
Source: Commun Biol. 2020 Aug 5;3:423. doi: 10.1038/s42003-020-01133-7 (PMC7406649; doi:10.1038/s42003-020-01133-7)
Supplement: Supplementary file 4 — Description of Additional Supplementary Files [file 42003_2020_1133_MOESM4_ESM.pdf]

Description of Additional Supplementary Files

File Name: Supplementary Data 1
Description: All sets of original data for main Figures, and original files of Supplementary Tables were included.
